# Metastatic Multiple Myeloma to the Skin

**DOI:** 10.1155/2019/7930123

**Published:** 2019-11-12

**Authors:** Alex C. Holliday, Mohammed I. Khan, Sean E. Mazloom, Rahul N. Chavan, Douglas J. Grider

**Affiliations:** ^1^Carilion Clinic and Virginia Tech Carilion School of Medicine, Department of Internal Medicine, Section of Dermatology, Roanoke, VA, USA; ^2^Alabama College of Osteopathic Medicine, Dothan, AL, USA; ^3^Virginia Tech Carilion School of Medicine, Department of Basic Science Education, Roanoke, VA, USA

## Abstract

Cutaneous involvement of multiple myeloma (MM) is uncommon, typically occurs in late stage disease, and is a poor prognostic indicator with an approximate eight month median survival. We present a 51-year-old man with relapsed lambda light chain MM who developed abrupt asymptomatic skin metastases. Biopsy revealed a dermis replete of atypical plasma cells, positive for CD138 and CD45. In situ hybridization confirmed lambda light chain restriction. Despite rescue antimyeloma therapy with the anti-CD38 drug daratumumab, he rapidly declined clinically and succumbed to the disease four weeks after presentation. A standard treatment approach for cutaneous MM does not currently exist; however, various techniques to detect cytogenetic abnormalities are emerging and will provide additional prognostic value and direct individualized therapy.

## 1. Case Presentation

**Figure 1:**
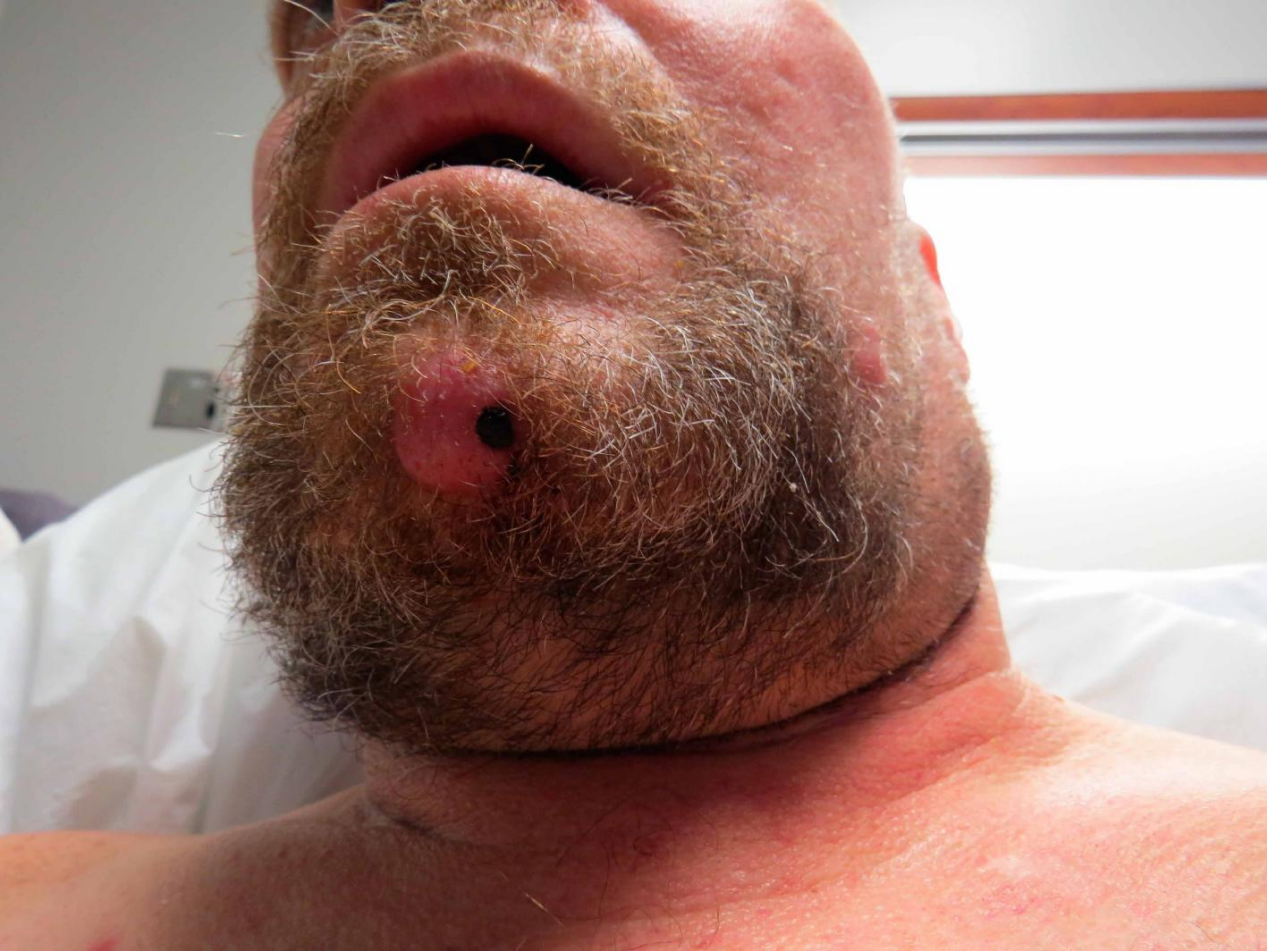
Physical examination revealed a pink nodule (with a 4 mm punch biopsy defect) on his chin and a pink plaque on his left cheek. Similar plaques and papules were evident on his upper trunk and scalp.

**Figure 2:**
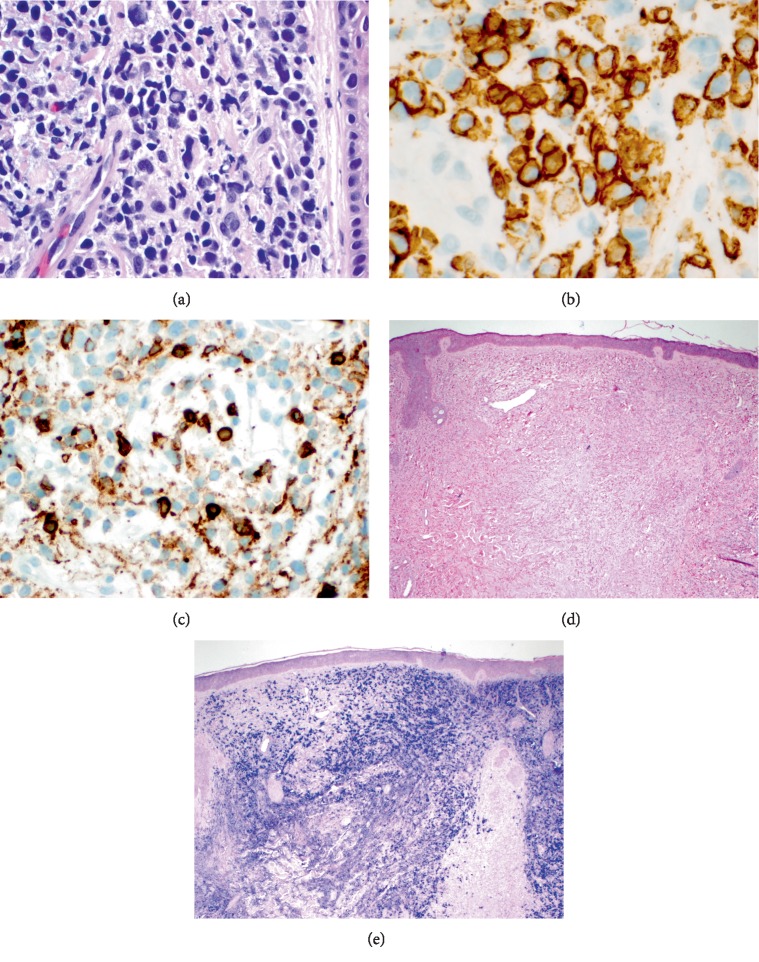
(a) Haematoxylin and Eosin stained tissue section from the chin punch biopsy. Atypical plasma cells with a Dutcher body apparent in the middle left (400 magnification; 40x). (b) CD138 positive (400 magnification, 40x). (c) CD45 positive (400 magnification, 40x). (d) Kappa light chain negative (40 magnification, 4x). (e) Lambda light chain positive (40 magnification, 4x).

A 51-year-old man with relapsed lambda light chain multiple myeloma (MM) was hospitalized due to uremic encephalopathy. Three years prior he experienced remission from MM after treatment with lenalidomide, bortezomib, and dexamethasone (RVD). At that time, he declined maintenance therapy and autologous stem cell transplant. After a relapse involving his cervical spine, he underwent de-compressive surgery, radiation, and restarted RVD. Subsequent bone marrow biopsy revealed disease progression. Dermatology was consulted regarding asymptomatic erythematous papules, plaques, and nodules involving his upper trunk, face, and scalp that had abruptly appeared over the prior three weeks (Figure 1). Punch biopsies from the chin nodule and a scalp plaque were obtained (Figure 2(a)). The papillary and reticular dermis were replete with atypical plasma cells, focally resembling plasmablasts, positive for CD138 (Figure 2(b)) and CD45 (Figure 2(c)) by immunohistochemistry. A Grenz zone underlined the epidermis and foci of malignant cells involving adnexal structures including pilosebaceous units were apparent. Dutcher bodies were rare; no Russell bodies were appreciated. In situ hybridization (ISH) confirmed lambda light chain restriction (Figures 2(d) and 2(e)).

The patient's renal failure was attributed to myeloma kidney; and hemodialysis, apheresis, and daratumumab were initiated. While his encephalopathy initially improved, the patient declined clinically and he was transitioned to comfort care three weeks after the biopsy, dying 5 days later.

## 2. Discussion

Multiple myeloma results from the accumulation of monoclonal protein producing plasma cells and principally affects the bone marrow. Common clinical manifestations are represented by the acronym CRAB: hyper*C*alcaemia, *R*enal insufficiency, *A*nemia, and *B*one lesions [1]. As of 2014, guideline revisions allow patients with a positive bone marrow biopsy and one CRAB feature or patients with smoldering myeloma and positive biomarkers predicting progression be offered treatment [1].

Cutaneous involvement of MM is uncommon. It usually occurs in late stage disease and reflects a high tumor burden. The median time from diagnosis to skin involvement is 2.2 years, but cutaneous MM may present at any time or in any stage of the disease [2]. There is an approximate eight month median survival after the appearance of cutaneous metastases [2, 3].

When skin lesions manifest in a MM patient, biopsy provides rapid assessment of disease severity. Histology is useful to differentiate direct tumor involvement from paraneoplastic processes like Sweet syndrome and complications from manufactured proteins such as those seen in amyloidosis [4].

Histopathologically, nodular and diffuse interstitial patterns exist. The neoplastic plasma cells typically stain strongly with CD79a, CD138, and epithelial membrane antigen; and variably with VS38c and CD43 [5]. Staining with CD138 (collagen-1 binding proteoglycan, syndecan) is particularly helpful as it is a specific marker for bone marrow derived plasma cells, both benign and malignant [5]. CD45 (leukocyte common antigen) immunohistochemical positivity is seen in relapsed MM [6]. Plasmablastic morphology can be misleading as it can mimic other blastic proliferations or be misinterpreted as a pseudolymphoma. A benign, dermal plasma cell rich proliferation can also be present at cutaneous-mucosal junctions or in special site areas like the scalp or the genitals. Thus, studies proving clonality, such as in situ hybridization for kappa and lambda light chains are important [2]. Light chain restriction does not occur in reactive plasma cell-rich infiltrates [3].

Acute renal insufficiency is a myeloma emergency, a poor prognostic indicator, and is attributed to the toxic effects of monoclonal light chains on the kidney [7]. Antimyeloma therapy coupled with aggressive hydration and avoidance of nephrotoxic agents is standard for acute renal insufficiency in MM patients [7].

Treatment and maintenance regimens include numerous combinations of traditional chemotherapy drugs, corticosteroids, proteasome inhibitors, immunomodulating agents, histone deacetylase inhibitors, interferon, monoclonal antibodies, and stem cell transplants. Although unsuccessful in our patient, the monoclonal antibody daratumumab targeting CD38 (a molecule overexpressed by MM cells) has been effective monotherapy in resistant and refractory disease [8].

## 3. Conclusion

With a dismal median survival, cutaneous MM confers a poor prognosis and treatment challenge for physicians. Research into cytogenetic abnormalities detected by fluorescent in situ hybridization is providing additional prognostic value and therapeutic application [9].
